# Impact of External Conditions on the Desorption and Degradation Capacity of Biochar for Rhodamine B

**DOI:** 10.3390/molecules30081717

**Published:** 2025-04-11

**Authors:** Chenghong Ao, Mai Shan, Yue Zhang, Xiang Li, Ying Kong, Xinwei Dong, Yang Liu, Danping Wu

**Affiliations:** 1 Yunnan Provincial Key Laboratory of Soil Carbon Sequestration and Pollution Control, Faculty of Environmental Science & Engineering, Kunming University of Science & Technology, Kunming 650500, China; chenghongaocd@163.com (C.A.); shanmai00616@163.com (M.S.); zy954280436@163.com (Y.Z.); m17809397653@163.com (X.L.); kongying81@163.com (Y.K.); dongxw@kust.edu.cn (X.D.); minipig6@163.com (Y.L.); 2 School of Energy and Environment Science, Yunnan Normal University, Kunming 650500, China

**Keywords:** biochars, rhodamine B, environmental implications, desorption, degradation, dissolved organic matter

## Abstract

Biochar has attracted considerable interest owing to its high adsorption capacity; however, the mechanisms through which environmental factors influence the release of adsorbed pollutants remain unclear. This study investigates the adsorption and desorption dynamics of Rhodamine B (RhB) on biochars B2 and B6, which were pyrolyzed at temperatures of 200 °C and 600 °C, respectively, under varying conditions. The results indicated that there was no significant difference in removal efficiency at low RhB concentrations; however, at a concentration of 600 mg/L, biochar B2 had a higher removal efficiency than B6, likely attributable to more adsorption sites. Increased temperatures were found to enhance desorption from both B2 and B6, with B6 exhibiting a faster desorption rate. This phenomenon may be due to the stronger hydrogen bonding between B2 and RhB, which could inhibit desorption. In addition, elevated pH values facilitated desorption, presumably through electrostatic repulsion. Under alkaline conditions, B2 released a greater amount of dissolved organic matter (DOM), leading to increased RhB desorption relative to B6. This study offers a valuable reference for evaluating the environmental risk associated with the application of biochar in real-world settings.

## 1. Introduction

Biochar is an ecofriendly and multifunctional material that serves as an effective adsorbent for eliminating both inorganic and organic contaminants [1,2,3]. Generally speaking, it is commonly utilized to remove heavy metals (such as Fe, Cu, Hg, As, and Pb) [4,5] and pesticides from soil [6] and organic dyes (such as Rhodamine B and Methyl blue) from water [7]. Biochar possesses a high specific surface area and favorable electron exchange capabilities, while its surface is rich in functional groups [8,9]. These characteristics allow biochar to quickly adsorb environmental contaminants, typically reaching equilibrium within a few hours to several tens of hours [10].

Until now, most studies have concentrated primarily on the contaminant removal process of biochar. For instance, biochar made from oil palm fronds can eliminate up to 93% of phenol and tannic acid within 900 min [11], with π-π interactions and hydrogen bonding being crucial in this process [12,13]. In addition, biochar made from Myriophyllum aquaticum can remove RhB through oxygen-containing functional groups, achieving equilibrium in just 72 h [14]. These findings confirm that biochar is an effective material for contaminant removal, with its surface structure playing a vital role in trapping contaminants. However, since the binding of biochar to contaminants largely relies on physical interaction, it is often susceptible to external environmental influences. For example, the phosphorus (P) sorption capacity of wheat-straw-derived biochar was influenced by pH levels [15]. As pH increases, P sorption decreases while desorption increases. Furthermore, the particle size of biochar also impacts sorption, with smaller particles enhancing the sorption capacity [16].

Generally, the experimental conditions employed in our studies are highly controlled and idealized. In contrast, real-world application conditions are influenced by a multitude of factors, including precipitation, temperature fluctuations, and variations in pH levels. Consequently, although the initial conditions of the application scenario may exhibit stability, they are subject to change due to environmental factors, such as diurnal temperature variations, seasonal shifts, and erosion caused by rainfall. Various external conditions may cause different alterations in the structure of biochar, including fragmentation and dissolution [17,18]. Previous studies have indicated that a decrease in pH can lead to reduced adsorption and increased desorption of contaminants [19,20]. This suggests that the changes occurring in this process could reduce the adsorption efficiency of biochar for contaminants, potentially resulting in secondary pollution due to the release of previously adsorbed contaminants. Moreover, the oxidation–reduction or degradation of the original contaminants may produce byproducts that may pose increased environmental hazards. Consequently, it is imperative to evaluate the desorption and degradation processes of pollutants from biochar under varying external conditions to inform the practical application of biochar.

In this study, Rhodamine B (RhB, C_28_H_31_ClN_2_O_3_), a common and harmful organic contaminant in water, was chosen as a model. Rice straw was selected as a raw material because it is the one of most productive agricultural wastes. Biochars were produced at temperatures of 200 °C and 600 °C, which were applied for removing varying initial concentrations of RhB. Following the attainment of equilibrium within the sorption system, subsequent desorption and degradation were analyzed under altered temperature and pH conditions. By correlating the specific surface area, distribution of functional groups, and inherent properties of the biochar with its desorption behavior, this study seeks to elucidate the mechanism through which external conditions affect biochar desorption.

## 2. Results and Discussion

### 2.1. Characterization of B2 and B6

**Figure 1:**
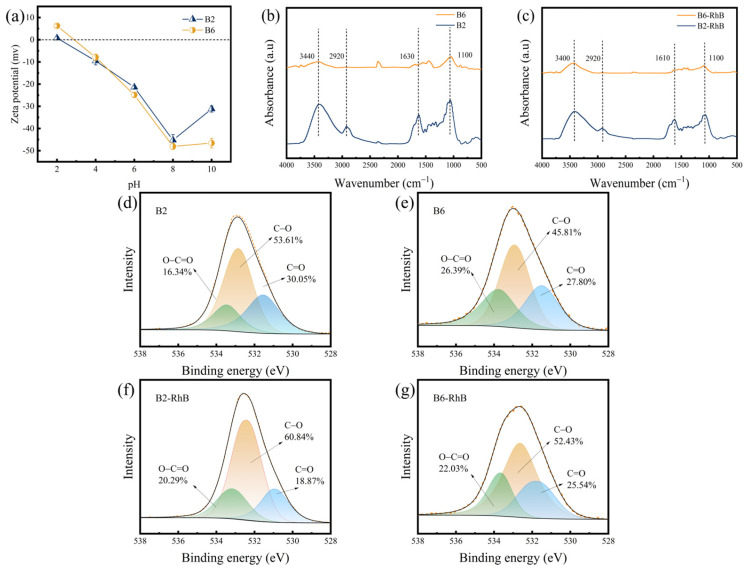
Characterization of B2 and B6. (**a**) Zeta potential of B2 and B6. (**b**) FTIR spectra of B2 and B6 before the adsorption of RhB. (**c**) FTIR spectra of B2 and B6 after the adsorption of RhB. (**d**) XPS of B2 before RhB adsorption. (**e**) XPS of B6 before RhB adsorption. (**f**) XPS of B2 after RhB adsorption. (**g**) XPS of B6 after RhB adsorption.

As known, the physicochemical properties of biochar, including functional groups, specific surface area, and surface charges, determine its removal capability for organic pollutants [21,22]. FTIR spectra were applied to acquire functional groups of biochars. As depicted in Figure 1b,c, both B2 and B6 showed characteristic peaks at 3400 cm^−1^ and 1100 cm^−1^, corresponding to the stretching vibration of -OH and C-O bonds, respectively [23]. Compared with B2, the -CH_2_- stretching vibration peak of B6 at 2850 cm^−1^ nearly disappeared, and its C=O stretching vibration peak at 1620 cm^−1^ weakened significantly, suggesting dehydrogenation and decarboxylation at high pyrolysis temperature. From XPS spectra, it can be seen that B2 and B6 contained peaks at 531.10 eV, 532.50 eV, and 534.04 eV (Figure 1d–g), corresponding to C=O, C-O, and O-C=O groups, respectively [24,25]. As the pyrolysis temperature increased, the content of Quinoid C=O and C-O slightly decreased, while the O-C=O proportion increased. This phenomenon can be ascribed to the intensified thermal decomposition of lignocellulosic components (e.g., hemicellulose, cellulose, and lignin) at higher temperatures, which may result in a structural reorganization of oxygen-containing moieties [26]. Elemental analysis of B2 and B6 was also conducted. B6 possessed lower hydrogen (H) and oxygen (O) contents than B2 (Table 1), which was attributed to decarboxylation, dehydrogenation, and condensation reactions during the high-temperature pyrolysis process. In addition, the O/C and (O+N)/C ratios of B6 were much lower than those of B2, showing an obvious reduction in oxygen-containing functional groups [27]. This was consistent with the FTIR data. That means that B6 had a lower polarity and hydrophilicity, leading to a weakening in its affinity for polar compounds and ionic molecules [28].

BET results showed that as the pyrolysis temperature increased from 200 °C to 600 °C (Table 1), the specific surface area of the biochar increased from 3.57 m^2^/g to 9.83 m^2^/g, resulting from volatilization and pore exposure [29]; additionally, the average pore diameter decreased from 15.99 nm for B2 to 13.44 nm for B6. SEM images also indicated that B2 exhibited a larger average pore size than B6, despite the fact that micropores and mesopores were not distinguishable at this resolution (Figures S1 and S2).

The surface charges of the as-prepared biochars were analyzed by zeta potential measurement. As depicted in Figure 1a, the zero point of charge (pHzpc) of B2 was approximately 2.04, while that of B6 was around 2.91. When the environmental pH is below the pHzpc, the functional groups on the biochar surface become protonated, showing a positive charge. Conversely, when the pH exceeds the pHzpc, the biochar surface undergoes deprotonation, exhibiting a negative charge [30].

### 2.2. RhB Removal Kinetics of B2 and B6

**Figure 2:**
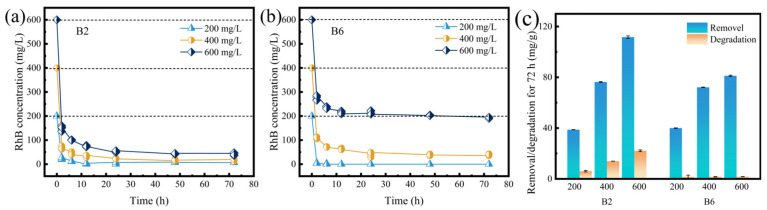
Removal and degradation of RhB by biochar at initial concentrations of 200, 400, and 600 mg/L. (**a**) Time-dependence curves of B2 for removal of RhB at initial concentrations of 200, 400, and 600 mg/L. (**b**) Time-dependence curves of B6 for removal of RhB at initial concentrations of 200, 400, and 600 mg/L. (**c**) The removal and degradation amounts of B2 and B6 for RhB at initial concentrations of 200, 400, and 600 mg/L.

When the initial concentration of RhB was 200 mg/L, both B2 and B6 removed almost all RhB within approximately 12 h (Figure 2a,b). When the initial RhB concentration increased to 400 mg/L, they took approximately 24 h to remove 90% of RhB in the solution. However, at 600 mg/L, the RhB removal rate of B2 remained ~90%, while that of B6 decreased to ~67% (Table S1). Note that the removal capacity of the as-prepared biochars outperformed that of many reported materials (Table S2) [31,32,33,34,35,36]. Moreover, the favorable removal capacity of B2 biochar is not limited to RhB but extends to various organic dyes. As illustrated in Table S2 and Figure S3, B2 achieves remarkable removal efficiencies for several dyes at high concentrations (1000 mg/L), with adsorption capacities of 191.84, 207.80, 100.76, and 90.57 mg/g for Methylene blue (MB), Basic fuchsin (BF), Methyl orange (MO), and Congo red (CR), respectively. Notably, B2 exhibits superior removal efficacy for cationic dyes in comparison with their anionic counterparts. This phenomenon was likely attributable to the electrostatic interactions between the negatively charged surface functional groups of B2 and the positively charged dye molecules (Table S3 and Figure S3).

**Figure 3:**
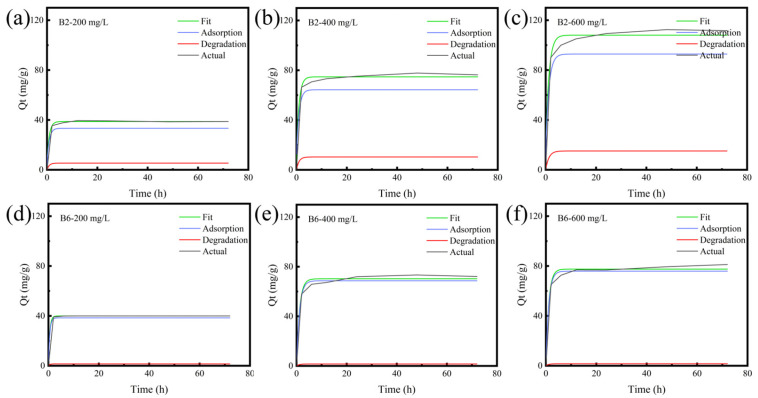
Using a two-compartment first-order model (2-PFOM) to analyze the adsorption and degradation of RhB by biochars. (**a**–**c**) The actual removal curves and 2-PFOM fitting curve of B2 for RhB at initial concentrations of 200, 400, and 600 mg/L, respectively. (**d**–**f**) The kinetic curves and 2-PFOM fitting of B6 for RhB removal at different initial concentrations.

Considering that biochar is able to degrade RhB, the RhB degradation amounts of B2 and B6 were determined. As shown in Figure 2c and Table S1, the RhB degradation amount of B2 was greater than that of B6. This can be attributed to the stronger catalytic and redox capability of B2. Specifically, the abundant functional groups on B2’s surface serve as mediators for electron transfer between the biochar and RhB, contributing to the catalytic degradation of RhB [37]. On the other hand, the phenolic groups in low-temperature biochar (B2) can act as electron donors to participate in redox reactions [38,39]. As the RhB concentration increased from 200 mg/L to 600 mg/L, the RhB degradation ratio of B2 rose from 15.66% to 19.62%. This may have been due to more sufficient contact between the biochar and RhB owing to the higher RhB concentration promoting the diffusion of RhB molecules into biochar micropores [40,41]. To analyze the contributions of adsorption and degradation, a two-compartment first-order model was used to fit the RhB removal, adsorption, and degradation data. As illustrated in Figure 3 and Table S4, the fitted curves matched well with the actual RhB removal curves of B2 and B6, with R^2^ values greater than 0.99. The adsorption rate constant k_1_ was one order of magnitude higher than the degradation rate constant k_2_, indicating that adsorption was the primary mechanism for RhB removed by B2 and B6. However, there was obvious degradation in the removal process, and the value of k_1_ could not accurately reflect the specific adsorption situation.

### 2.3. RhB Adsorption of B2 and B6

The amounts of RhB adsorbed on biochars with time were obtained according to the two-compartment first-order model results. Specifically, the amounts of RhB adsorbed on biochars at different intervals were calculated by subtracting the fitting degradation amount from actual removal amounts. Isothermal adsorption experiments were also conducted at various RhB initial concentrations (50–600 mg/L). Once adsorption equilibrium was achieved, the adsorbed RhB was extracted from biochars using acetonitrile to determine their equilibrium adsorption capacities (Q_e_).

As presented in Tables S5 and S6, when the initial RhB concentration increased, the amount of RhB adsorbed on biochars at equilibrium significantly increased. At 600 mg/L, B2 exhibited a higher Q_e_ value than B6. This may have been due to the surface of B2 being rich in oxygen-containing functional groups, which can form hydrogen bonds with RhB molecules for enhancing adsorption [42]. RhB, a cationic dye with amino groups and benzene rings, can adsorb onto biochar via hydrogen bonding, π-π interactions, and electrostatic attraction. B6 likely adsorbs RhB onto its carbonized surface because of its larger specific surface area and porous structure. In contrast, B2 may exhibit both surface adsorption and the participation of residual organic matter [43].

**Figure 4:**
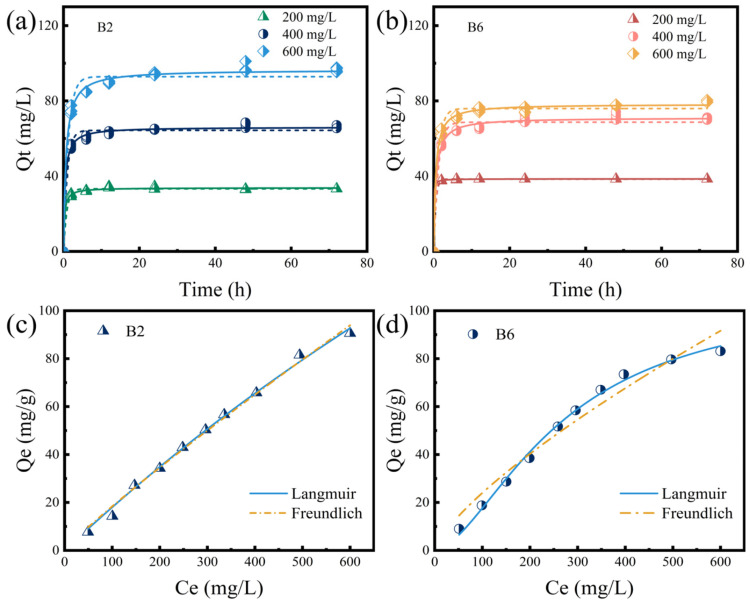
Fitting of RhB adsorption by biochar B2 and B6. Adsorption kinetic curves of (**a**) B2 and (**b**) B6 for RhB at different initial concentrations fitted with a PFOM (dashed line) and PSOM (solid line) model. (**c**,**d**) Adsorption isotherm fitting of B2 and B6, respectively, to RhB.

The kinetic profiles exhibited a rapid rise in the early stage and then quickly reached a plateau. At the beginning of adsorption, a large concentration gradient between the solid phase (adsorbed RhB on biochar) and the aqueous phase (unadsorbed RhB in the solution) provided a strong driving force for the mass transfer of RhB [40]; meanwhile, the biochar surface contained numerous unoccupied adsorption sites [44], facilitating the rapid adsorption of RhB [45]. With the progress of adsorption, the concentration gradient decreased gradually, and the pores and adsorption sites of the biochar became saturated [46], resulting in a slower adsorption rate until the equilibrium [47]. As shown in Figure 4 and Table S4, the adsorption data were further fitted with PFOM and PSOM models. The PSOM model was better fitted with the RhB adsorption kinetics of B2 and B6 at different RhB concentrations, and the calculated Q_e_ values matched well with the experimental values (Figure 4 and Table S4), suggesting the adsorption process of biochar for RhB was primarily governed by chemical adsorption accompanied by physical adsorption. In addition, both B2’s and B6’s adsorption isotherm data matched well with a Langmuir model (R^2^ = 0.994–0.995, Table S5). These results suggested the monolayer adsorption of RhB on B2 and B6.

After RhB adsorption, the -OH stretching peak of biochar shifted from 3440 cm^−1^ to 3400 cm^−1^ (Figure 1c), indicating the formation of hydrogen bonding between -OH groups on biochar and RhB [48]. Additionally, the bending vibration peak of aromatic C=C shifted from 1630 cm^−1^ to 1610 cm^−1^ (Figure 1c), supporting π-π interaction between the biochar’s benzene rings and RhB [49]. XPS analysis was used to examine changes in the elemental composition and bonding states of biochar during RhB adsorption (Figure 1d–g). Quinoid C=O may act as an electron acceptor in redox reactions [20,47], showing decreased content in all biochars after RhB adsorption. This result further implied the chemical adsorption of B2 and B6 for RhB.

### 2.4. The Desorption of RhB on B2 and B6 at Different Conditions

**Figure 5:**
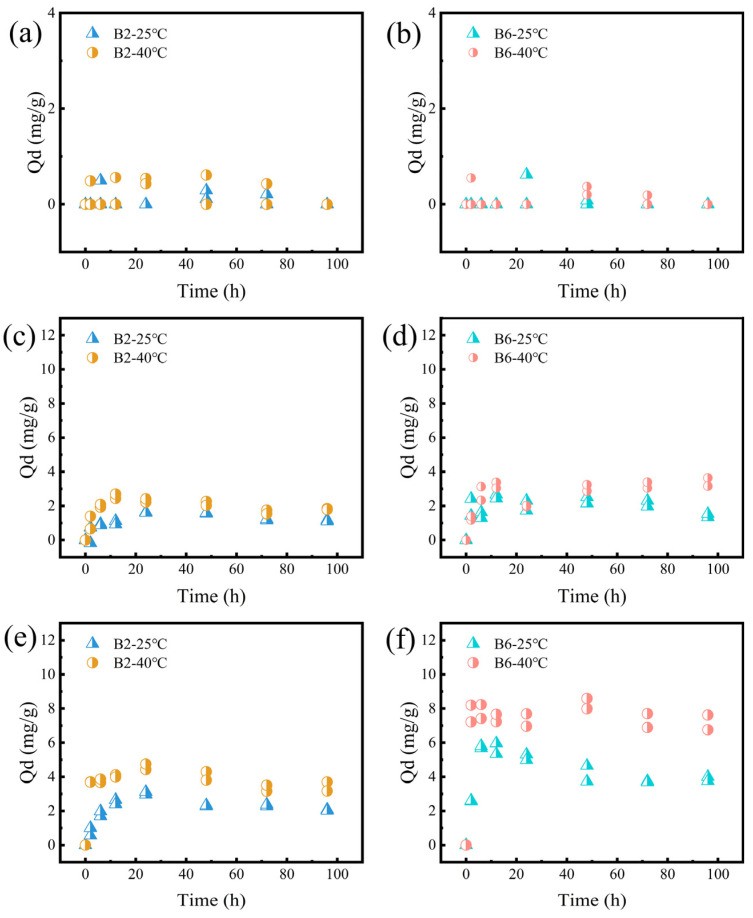
Dynamic RhB desorption data of B2 at 25 °C and 40 °C, with initial RhB concentrations of (**a**) 200, (**c**) 400, and (**e**) 600 mg/L. Desorption kinetics of B6 at 25 °C and 40 °C, with initial RhB concentrations of (**b**) 200, (**d**) 400, and (**f**) 600 mg/L.

After the RhB adsorption of biochars at different initial concentrations reached equilibrium, desorption experiments were conducted by adding ultrapure water (Figure 5). At 200 mg/L of RhB, no significant desorption was observed. However, at 400 mg/L and 600 mg/L, an increase in the desorption amount occurred. This phenomenon can be ascribed to the heterogeneous structure of biochar. Specifically, biochar with a heterogeneous structure possesses at least two types of active sites: high-energy and low-energy sites [50,51]. This heterogeneity may leads to biphasic or multiphasic desorption kinetics [52]. Molecules adsorbed on low-energy sites were easier to desorb than those on high-energy sites because the latter had the stronger affinity toward adsorbed molecules. When the initial RhB concentration increased, the high-energy sites were occupied at first, and then more RhB molecules adsorbed onto the low-energy sites, resulting in easy desorption under external disturbances.

#### 2.4.1. Effect of Temperature

The desorption behavior at different temperatures (25 °C and 40 °C) is shown in Figure 5. At 40 °C, the amount of RhB desorbed from biochar increased. This phenomenon may have been due to the violent thermal motion at the higher temperature overcoming the desorption energy barrier [41,53]. Under the same conditions, the RhB amount desorbed from B6 was higher than that from B2, which can be attributed to the fact that B2 contained more abundant functional groups. These functional groups may facilitate the formation of electrostatic interactions and hydrogen bonding, making it more difficult for RhB to desorb from B2 than from B6.

#### 2.4.2. Effect of pH

**Figure 6:**
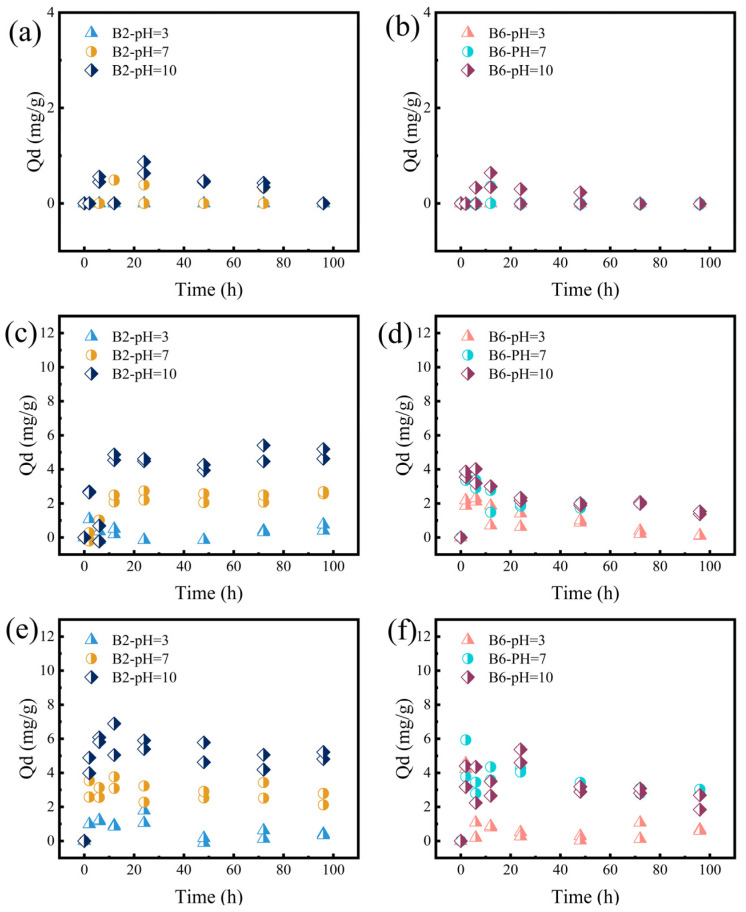
Effects of pH values on the desorption of B2 at the initial RhB concentrations of (**a**) 200, (**c**) 400, and (**e**) 600 mg/L. Influences of pH values on the desorption of B2 for RhB after adsorption of RhB for 72 h at the initial concentrations of (**b**) 200, (**d**) 400, and (**f**) 600 mg/L.

The desorption behavior of RhB from biochar under different pH conditions (pH = 3, 7, 10) is shown in Figure 6. The zeta potential results indicated that biochar exhibited a negative charge across the pH range of 3–10, which was consistent with numerous studies [54,55]. After deprotonation of hydroxyl and carboxylic groups on biochar surface, the competition between H^+^ and cations is minimized, facilitating the adsorption of cationic species [30,56]. As a cationic dye, RhB exists primarily in the form of a tertiary amine cation and molecular under acidic conditions (pH < 4.0) [53]. When the pH exceeds 4.0, the concentration of RhB cations decreases significantly, and the zwitterionic form of RhB^±^ becomes dominant, leading to the aggregation of RhB molecules. At pH = 8.0, the increased negative charge and excess OH^−^ ions cause competition between -COO- and -N^+^ groups, inhibiting the aggregation of RhB [57]. Therefore, under pH = 3 conditions, the biochar was negatively charged, and RhB was positively charged, so that electrostatic attraction between them inhibited the desorption of RhB. In contrast, at pH = 7 and 10, the increased negative charge of RhB enhanced electrostatic repulsion, leading to the higher amount of RhB desorbed from the biochar. Moreover, B2 rather than B6 contained a certain proportion of DOM due to incomplete carbonization or the generation of small molecular components at low pyrolysis temperatures [58]. Under alkaline conditions, DOM was easy to leach, accompanied with adsorbed RhB, which led to the highest desorption amount at pH = 10 (Table S7). The highest DOM leaching at pH = 10 and the lower RhB desorption amount from B2W further confirmed this assumption (Figure S4).

### 2.5. RhB Degradation of Biochars

**Figure 7:**
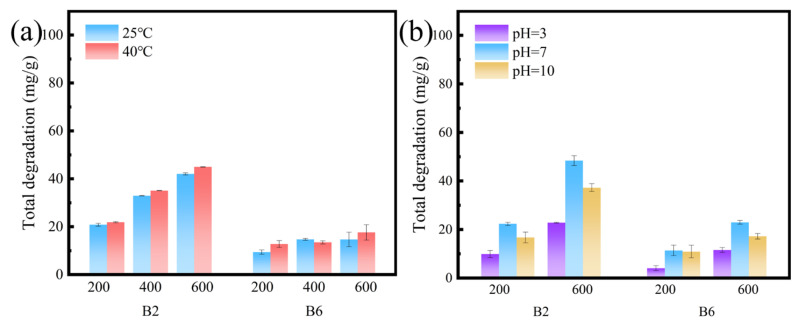
The RhB degradation of B2 and B6 during the adsorption process. (**a**) Desorption at 25 °C and 40 °C with initial concentrations of RhB at 200, 400 and 600 mg/L. (**b**) Desorption at different pH (pH = 3, 7, 10) with initial concentrations of RhB at 200 and 600 mg/L.

The degradation of RhB by biochar under various conditions is shown in Figure 7. The degradation process initiates on the biochar’s surface, subsequently progressing as RhB diffusion into the interior of the biochar, where it interacts with internal functional groups [59]. Overall, B2 exhibited a higher degradation amount than B6 (Table S1).

External conditions also affect the degradation process. At 40 °C, the degradation amount was slightly higher than that observed at 25 °C, which was likely attributable to enhanced molecular motion that facilitated the diffusion of RhB into the pores of the biochar and promoted reactions with active species. The pH level can influence RhB degradation by affecting the degradation pathway [60]. The primary mechanism of degradation involves N-demethylation, exhibiting reduced efficiency at both very low and high pH levels. Hydrochloric acid (HCl) and sodium hydroxide (NaOH) were employed to modulate the pH. At low pH levels, the increased concentration of Cl^−^ ions may neutralize H^+^ ions and hydroxyl radicals, while at high pH levels, OH^−^ ions may absorb hydroxyl radical-producing holes, thereby inhibiting the degradation process [61].

## 3. Materials and Methods

### 3.1. Materials

Rhodamine B (RhB), sodium hydroxide (NaOH, ≥99%), and hydrochloric acid (HCl, 37%) were purchased from Aladdin (Shanghai, China). Methanol (CH_3_OH, ≥99.9%) and ethanol (C_2_H_5_OH, ≥99.9%) were obtained from Sigma Aldrich (Burlington, MA, USA). Rice straw was purchased from the Lianyungang Surui agricultural products deep processing company (Lianyungang, China). Ultrapure water was used throughout experiments.

### 3.2. Biochar Preparation

The rice straw was pyrolyzed under N_2_ atmosphere in a tube furnace (SK-Q15123K-610, China) at 200 °C and 600 °C for 4 h, with a heating rate of 10 °C min^−1^, and then cooled to room temperature with a rate of 5 °C min^−1^. The resulting biochars were screened using a 100-mesh sieve and denoted as B2 and B6, representing the different maximum pyrolysis temperatures. A part of B2 was washed with ultrapure water to remove dissolved organic matter (DOM) and then named as B2W.

### 3.3. Characterization

Elemental compositions of samples were measured with an element analyzer (EA, Flashsmart CHNS-Thermo Fisher, Waltham, MA, USA). N_2_ adsorption/desorption isotherms of samples at 77 K were studied using a Brunauer–Emmett–Teller analyzer (BET, ASAP 2020-Micromeritics, Norcross, GA, USA), which was applied to determine their specific surface area and pore volume. Prior to testing, the samples were dried at 120 °C for 12 h. An X-ray photoelectron spectrometer (XPS, Thermo Scientific K-Alpha, Waltham, MA, USA) and a Fourier-transform infrared spectrometer (FTIR, Varian640-IR, Palo Alto, CA, USA) were applied to evaluate the functional groups of the as-prepared biochar before and after adsorption of RhB. FTIR measurement was carried out in the range of 4000–400 cm^−1^ with a resolution of 4 cm^−1^. XPS analysis was conducted using the Avantage-5.9931 software. Zeta potential measurements were performed for each sample in a Zetasizer Nano Series (Zeta, Malvern Zetasizer Nano ZS90, Malvern, UK). The concentrations of RhB were determined by high-performance liquid chromatography (HPLC, Agilent-1260, Santa Clara, CA, USA) at a wavelength of 550 nm. A mixture of methanol and water (75:25, *v/v*) was chosen as a mobile phase, and the flow rate was 1 mL/min.

### 3.4. Batch Adsorption and Desorption Experiments

A series of RhB aqueous solution (200 mg/L, 400 mg/L, and 600 mg/L) were prepared by diluting 800 mg/L RhB stock solution with ultrapure water. Of this RhB aqueous solution, 20 mL was mixed with 100 mg of biochars in brown glass vials and then placed in a shaker (ZH-D Jingda Instrument manufacturing compay, Jintan, Jiangsu, China) in the dark. After shaking for 2 h, 6 h, 12 h, 24 h, 48 h, and 72 h, the biochars were centrifuged at 3000 rpm for 15 min, and the supernatants were measured using HPLC.

To simulate desorption processes under varying environmental conditions, the samples after adsorption of RhB for 72 h were centrifuged at first; after pouring out the supernatant, ultrapure water with different temperatures (25 °C, 40 °C) and various pH levels (pH = 3, 7, 10) was added; then, the bottles containing biochar and ultrapure water were placed on a shaker at a speed of 120 r/min. At preset intervals, each solution was centrifuged, and the supernatant was withdrawn for HPLC measurement. By contrast, the adsorption experiment at a constant condition was conducted as a control group.

To distinguish the degradation and adsorption of RhB on biochar, the RhB amount adsorbed on the biochar was measured using an oscillating solvent extraction method [38]. In brief, after adsorption/desorption equilibrium, the supernatant was removed by centrifugation, and 20 mL of acetonitrile was added to the residual biochar solid particles; then, it oscillated at 120 r/min for 2 h to ensure the sufficient extraction of RhB. This procedure was repeated until the RhB concentration in the acetonitrile below the HPLC detection limit. For all removal, adsorption, and degradation experiments, triplicate samples were prepared and analyzed, with the mean values reported as the experimental results. The RhB adsorption amount on the biochar was calculated based on the extraction amount in the acetonitrile. The RhB degradation amount was calculated by subtracting the adsorption amount from the total removal amount [38]:

(1) $$Q_{removal}=Q_{ads}+Q_{deg}$$

where Q_removal_ (mg/g) is the total removal amount of the pollutant and Q_ads_ (mg/g) and Q_deg_ (mg/g) are the pollutant removal amounts via adsorption and degradation, respectively.

### 3.5. Data Analysis

In this experiment, the two-compartment first-order sorption model (2-PFOM) [62] was employed to simulate the removal, adsorption, and degradation processes of RhB by B2 and B6. The adsorption mechanisms of biochar toward RhB were systematically investigated through equilibrium isotherm analysis and kinetic modeling. Specifically, the Langmuir [63] and Freundlich [64] isotherm models, along with the pseudo-first-order (PFSO) [65] and pseudo-second-order (PSOM) [42,66] kinetic models, were applied. The corresponding mathematical equations, characteristic parameters, and correlation coefficients for both isotherm and kinetic analyses are comprehensively summarized in Tables S4 and S5.

A two-compartment first order sorption model (2-PFOM) was utilized to simulate the removal process of RhB, and the formula is provided below:

(2) $$\frac{Q_{t}}{Q_{e}}=f_{1}\left(1-e^{{-k}_{1}t}\right)+f_{2}\left(1-e^{-k_{2}t}\right)$$

where Q_t_ (mg/g) and Q_e_ (mg/g) are sorbate concentration in the solid phase at time t and adsorption equilibrium, respectively; additionally, k_1_ and k_2_ represent the adsorption and degradation rate constants, respectively, while f_1_ and f_2_ represent the fractions of the two compartments, f_1_ + f_2_ = 1. Since the adsorption and degradation fractions are quantitatively determined at the end of the reaction, f_1_ and f_2_ are identifiable parameters.

The Langmuir model was used to characterize monolayer adsorption on homogeneous surfaces, which assumes identical binding sites without intermolecular interactions. The corresponding formula is provided as follow:

(3) $$Q_{e}=\frac{Q_{m}K_{L}C_{e}}{1+K_{L}C_{e}}$$

The Freundlich model was applied to describe multilayer adsorption on heterogeneous surfaces, reflecting empirical fitting of nonideal systems. The nonlinear form of the equation is expressed as follow:

(4) $$Q_{e}=K_{F}C_{e}^{\frac{1}{n}}$$

where Q_e_ (mg/g) is the equilibrium adsorption capacity, Q_m_ (mg/g) represents the theoretical maximum monolayer adsorption capacity, C_e_ (mg/L) denotes the equilibrium concentration of RhB in solution, K_L_ is the Langmuir equilibrium constant related to adsorption affinity, K_F_ represents the Freundlich constant associated with adsorption capacity, and n is the heterogeneity factor characterizing surface energetics.

The pseudo-first-order model (PFOM) was used to examine the effects of surface diffusion, which is associated with physical adsorption. The corresponding formula is provided:

(5) $$Q_{t}=Q_{e}*\left(1-e^{-k_{1}t}\right)$$

The pseudo-second-order model (PSOM) was employed to analyze the chemisorption process occurring between the adsorbent and the adsorbate, with the relevant formula presented as follows:

(6) $$Q_{t}=\frac{{Q_{e}}^{2}*k_{2}t}{1+k_{2}t}$$

where Q_t_ (mg/g) and Q_e_ (mg/g) are concentrations in solid phase at time t and adsorption equilibrium, respectively; k_1_ is the rate constant for pseudo-first-order adsorption; and k_2_ is the rate constant for pseudo-second-order adsorption.

## 4. Conclusions

Biochars prepared at different temperatures exhibited distinct physicochemical properties, resulting in variations in their adsorption capability. The low-temperature biochar (B2) had a lower specific surface area compared with high-temperature biochar (B6), but B2 exhibited higher adsorption and degradation capacities owing to its richer functional groups. Furthermore, B2 and B6 showed different RhB desorption behaviors under various external conditions. The RhB desorption amount of B2 was lower than that of B6 even at the elevated temperature, which was due to its abundant functional groups. The desorption variations in various pH values were more obvious than those in different temperatures. Under acidic conditions, both B2 and B6 exhibited minimal desorption amounts due to the electrostatic interactions between biochar and RhB molecules. Conversely, under alkaline conditions, a high amount of DOM released from B2, leading to a clear increase in the desorption amount. This work provides insight into the secondary release of adsorbed RhB at different environment conditions.

## Figures and Tables

**Table 1:** Elemental data and specific surface area of as-prepared biochars.

Analysis	Unit	B2	B6	B2W
C	(%)	46.26	61.16	48.75
H	(%)	4.97	1.35	5.40
O	(%)	33.40	3.57	36.16
N	(%)	1.03	0.58	0.94
H/C	-	0.11	0.02	0.11
O/C	-	0.72	0.06	0.74
(N+O)/C	-	0.74	0.07	0.85
Surface area	(m^2^/g)	3.57	9.83	3.67
Micropore area	(m^2^/g)	1.91	5.70	1.08
Pore volume	(cm^3^/g)	0.014	0.033	0.014
Average pore diameter	(nm)	15.99	13.44	15.63

## Data Availability

The data presented in this study are available upon request from the corresponding author. The data are not publicly available due to privacy reasons.

## Supplementary Materials

The following supporting information can be downloaded at: https://www.mdpi.com/article/10.3390/molecules30081717/s1, Figure S1: SEM images of B2 (a–d) and B6 (e–h); Figure S2: The average diameter and size distributions of (a) B2 and (b) B6; Figure S3: The removal amount of Methylene blue (MB), Basic fuchsin (BF), Methyl orange (MO), and Congo red (CR) by B2; Figure S4: Dynamic desorption data of B2W at pH=10, with different RhB initial concentrations; Table S1: The removal and degradation data of RhB by B2 and B6; Table S2: Comparison of adsorption capacity between biochars and other adsorbents; Table S3: The removal amount of Methylene blue (MB), Basic fuchsin (BF), Methyl orange (MO), and Congo red (CR) by B2; Table S4: 2-PFOM fitting parameters for the RhB removal using B2 and B6; Table S5: Adsorption kinetic parameters for the RhB adsorption on B2 and B6; Table S6: Adsorption isotherm parameters for the RhB adsorption on B2 and B6; Table S7: DOM release concentration under different conditions.

## Abbreviations

The following abbreviations are used in this manuscript:

RhB	Rhodamine B
DOM	Dissolved organic matter
