# Supplementary material for: Impact of External Conditions on the Desorption and Degradation Capacity of Biochar for Rhodamine B
Source: Molecules. 2025 Apr 11;30(8):1717. doi: 10.3390/molecules30081717 (PMC12029349; doi:10.3390/molecules30081717)
Supplement: Supplementary file 1 [file molecules-30-01717-s001.zip › molecules-3526376-supplementary.pdf]

Article

Impact of External Conditions on the Desorption and Degradation Capacity of Biochar for Rhodamine B

Chenghong Ao [1], Mai Shan [1], Yue Zhang [1], Xiang Li [1], Ying Kong [1], Xinwei Dong [1], Yang Liu [1] and Danping Wu [2,*]

[1] Yunnan Provincial Key Laboratory of Soil Carbon Sequestration and Pollution Control, Faculty of Environmental Science & Engineering, Kunming University of Science & Technology, Kunming 650500, China; chenghongaocd@163.com (C.A.); shanmai00616@163.com (M.S.); zy954280436@163.com (Y.Z.); m17809397653@163.com (X.L.); kongying81@163.com (Y.K.); dongxw@kust.edu.cn (X.D.); minipig6@163.com (Y.L.)

[2] School of Energy and Environment Science, Yunnan Normal University, Kunming 650500, China

* Correspondence: wudp240005@163.com

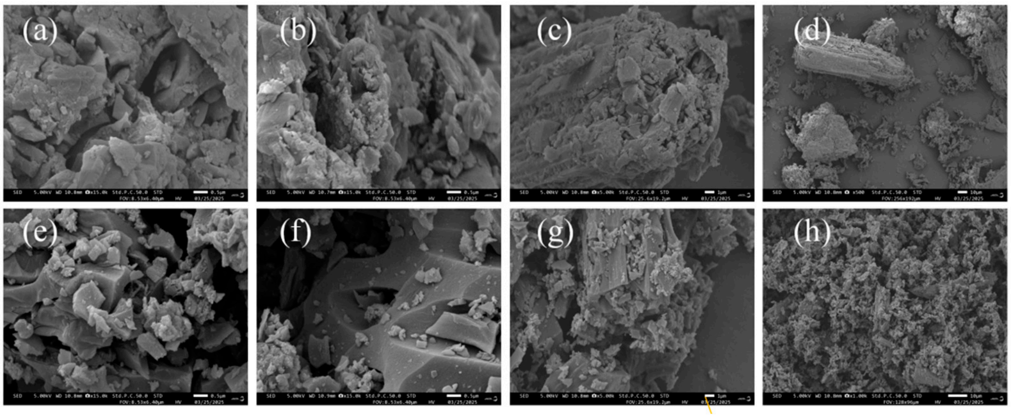

Figure S1. SEM images of B2 (a-d) and B6 (e-h).

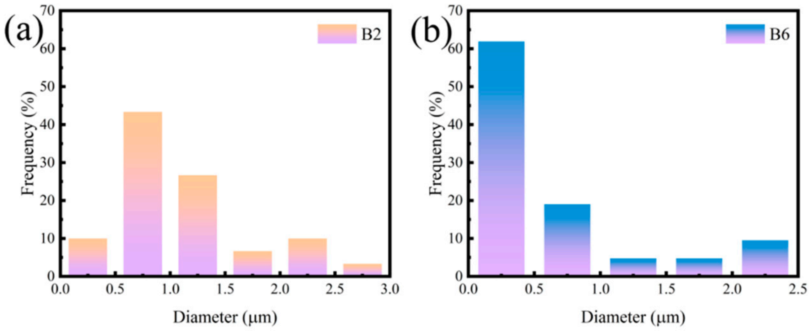

Figure S2. The average diameter and size distributions of (a) B2 and (b) B6.

Table S1. The removal and degradation data of RhB by B2 and B6.

Sample	RhB initial concentration (mg/L)	RhB removal amount (mg/g)	RhB degradation amount (mg/g)	Degradation/ removal amount (%)
B2	200	38.76	6.07	15.66

	400	76.34	13.97	18.41
	600	111.65	22.02	19.62
	200	40	1.62	2.25
B6	400	72.14	1.64	2.03
	600	81.22	1.72	2.12

Table S2. Comparison of adsorption capacity between biochars and other adsorbents.

Pollutant type	Materials	Targeted pollutants	Maximum adsorption capacity (mg/g)	References
Heavy metals	Sludge biochar supported nanoscale zero-valent iron	Cd^{2+}, Cu^{2+}	Cd^{2+}: 55.94, Cu^{2+}: 97.68	International journal of environmental research and public health, 2022, 16041
	Biochar from muskmelon peel	Cu^{2+}, Zn^{2+}	Cu^{2+}: 78.74, Zn^{2+}: 72.99	Bioresource technology, 2022, 126425.
	Modified biochar from rice husk	Cd^{2+}, Cu^{2+}	Cd^{2+}: 104.34, Cu^{2+}: 125.34	
	Activated carbon	Cd^{2+}, Pb^{2+}	Cd^{2+}: 17.23, Pb^{2+}: 16.84	Journal of environmental chemical engineering, 2017,679-698
Dyes	Biochar from durian rinds	Indigo carmine	192.55	Case studies in chemical and environmental engineering, 2024, 100997
	Ziziphus jujuba waste-derived biochar	Indigo carmine	166.46	Desalination and water treatment, 2023, 258-270
	Mg/Fe layered double hydroxide nanoparticles	Indigo carmine	55.5	Chemosphere, 2017,280-288
	Rise-ask ash	Indigo carmine	65.59	

Table S3. The removal amount of Methylene blue (MB), Basic fuchsin (BF), Methyl orange (MO), and Congo red (CR) by B2.

Sample	RhB initial concentration (mg/L)	Removal amount (mg/g)			
		MB	BF	MO	CR
B2	1000	191.84	207.80	100.76	90.57

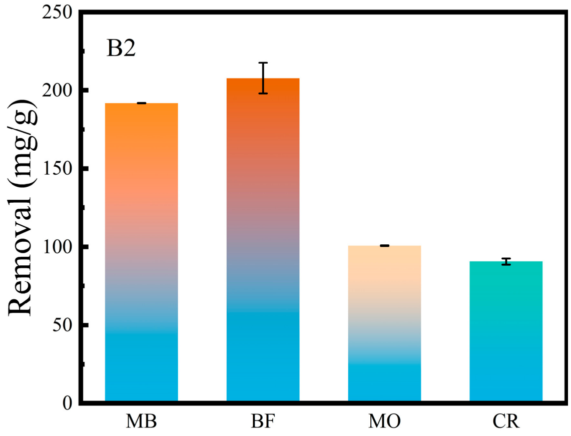

Figure S3. The removal amount of Methylene blue (MB), Basic fuchsin (BF), Methyl orange (MO), and Congo red (CR) by B2.

Table S4. 2-PFOM fitting parameters for the RhB removal using B2 and B6.

Sample	RhB initial concentration (mg/L)	Actual removal amount (mg/g)	Fitted removal amount (mg/g)	2-PFOM		
				k_1	k_2	R^2
B2	200	38.76	38.73	26.59	1.15	0.997
	400	76.34	74.72	19.98	1.00	0.992
	600	111.65	108.01	23.20	0.78	0.989
B6	200	40	39.98	216.93	1.93	0.999
	400	72.14	70.28	294.83	0.84	0.989
	600	81.22	78.56	311.19	0.89	0.991

Table S5. Adsorption kinetic parameters for the RhB adsorption on B2 and B6.

Sample	RhB concentration (mg/L)	Adsorption(mg/g)	PFOM			PSOM		
			k_1	Q_e	R^2	k_2	Q_e	R^2
B2	200	33.31	1.15	33.31	0.996	0.12	33.31	0.997
	400	65.96	1.01	64.34	0.991	0.04	66.08	0.996
	600	96.52	0.80	92.87	0.985	0.02	96.55	0.996
B6	200	38.48	1.93	38.46	0.999	0.56	38.47	0.999
	400	70.55	0.84	68.69	0.988	0.03	71.09	0.996
	600	79.59	0.89	75.93	0.991	0.03	78.30	0.997

Table S6. Adsorption isotherm parameters for the RhB adsorption on B2 and B6.

Sample	Langmuir			Freundlich		
	k_L	Q_m	R^2	k_F	1/n	R^2
B2	3.41	545.60	0.995	0.28	0.91	0.993
B6	9.43	107.44	0.994	0.77	0.75	0.954

Table S7. DOM release concentration under different conditions.

Different conditions	DOM concentration (mg/g)	
	**B2**	**B6**
Dark	3.22	0
Light	11.32	0.1161
25℃	3.27	0
40℃	7.25	0
pH=3	1.74	0.27
pH=7	3.93	0
pH=10	32.38	0.28

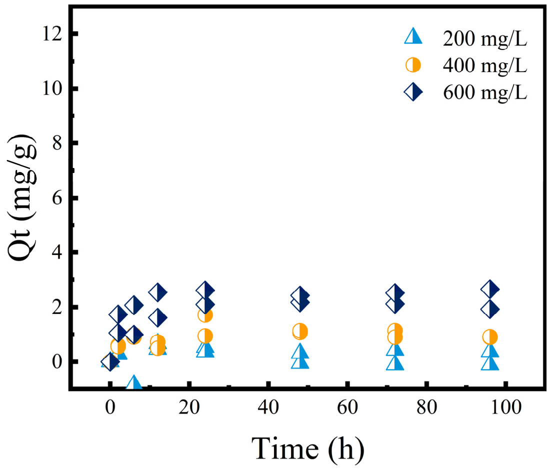

Figure S4. Dynamic desorption data of B2W at pH=10, with different RhB initial concentrations.
